# Ambient ageism: Exploring ageism in acoustic representations of older adults in AgeTech advertisements

**DOI:** 10.3389/fsoc.2022.1007836

**Published:** 2022-10-10

**Authors:** Megan E. Graham

**Affiliations:** ^1^Department of Sociology and Anthropology, Faculty of Arts and Social Sciences, Carleton University, Ottawa, ON, Canada; ^2^Department of Sociology, Trent University, Peterborough, ON, Canada

**Keywords:** AgeTech, smart home, ageing-in-place, sound, advertisement, ageism

## Abstract

Ageing-in-place environments are increasingly marked by ambient digital technologies designed to keep older adults safe while they live independently at home. These AgeTech companies market their products by constructing imagined visual and aural worlds of the smart home, usually deploying ageist representations of ageing and older adults. The advertisements are multimodal, and while what is seen on screen is often considered most important in a visuo-centric western culture, scholars have argued that it is what audiences hear that has the greatest impact. The acoustic domain of AgeTech advertisements and its relationship to ageism in marketing has not yet been explored. Accordingly, this paper will address this gap by following Van Leeuwen's framework for critical analysis of musical discourse to explore what AgeTech companies say about ageing, older adults, and ageing-in-place technologies using sound in an illustrative set of smart home advertisements for ageing-in-place. The paper will discuss how music, voice, and sound are semiotic resources that are used to construct stereotypical (both negative and positive) portrayals of older adults, reinforce the narrative of “technology as saviour,” and trouble the private/public boundaries of the ageing-in-place smart home.

## Introduction

The global population of older adults is increasing at an exceptional pace. According to the WHO, the world can expect to have 1.4 billion people aged 60 years and older by 2030, and 2.1 billion by 2050 (WHO, 2022a). Accompanying this incredible rise in the number of older adults is an urgent concern for creating supportive age-friendly societies through formal and informal modalities of care, transportation, housing, and city planning (WHO, 2022a). On an international level, there is recognition that healthy ageing—the combination of individual physical and mental capacity and physical, social, and systemic environments—needs to be addressed. The United Nations Decade of Healthy Ageing (2021–2030) has created an action plan that addresses age-friendly environments, integrated care, long-term care, and combatting ageism. Importantly, in relation to these action areas, the WHO website states that, “To foster healthy ageing and improve the lives of older people and their families and communities, fundamental shifts will be required not only in the actions we take but in how we think about age and ageing” (WHO, 2022b, n.pag.).

Increasingly, technology is seen as a key solution to creating environments for healthy ageing. Technology has been positioned as hero in an apocalyptic tale of silver tsunamis and an ever-greying planet for which societies are poorly equipped to manage with human labour alone (Higgs and Gilleard, 2022). AgeTech, technology designed specifically for older adults, simultaneously addresses and constructs ideas of age and ageing, so-called healthy and independent ageing environments. One of the defining features of healthy ageing (often synonymous with active ageing and successful ageing) is the capacity to age in place. Dalmer et al. (2022) note that ageing-in-place is a social, economic, and political objective wherein a person continues to live in their home and postpone moving into an institutional care setting for as long as possible. The authors add that “With changing demographics and geographically dispersed families, technologies in the home are promoted as ensuring greater autonomy and safety for “at risk” older adults as they age in place” (p. 84). Moreover, Marshall et al. (2022) analysed the values that accompany technologically assisted ageing-in-place in the Netherlands, Spain, and Canada, finding that the top values were quality of life, independence and autonomy, risk management, social inclusion, and active ageing.

The reliance on smart home technologies (specifically, passive monitoring technologies) is simultaneously helpful and fraught with problems. The debated issues include the ethics of ambient or passive monitoring systems (Pirzada et al., 2022), and the perception of in-home monitoring technology among older adults and the associated impact on intergenerational relationships (Berridge, 2016, 2017; Berridge and Westle, 2019; Tural et al., 2021). Scholars in the field of socio-gerontology, and more specifically, socio-gerontechnology, are applying a critical lens to technological solutions that support ageing-in-place (Peine et al., 2021; Urban, 2021; Van Hees et al., 2021), including for older adults living with dementia (Vermeer et al., 2019, 2020).

Accompanying these processes of design and innovation, researchers are also pointing to the ageist discourses that are invoked in the marketing of these technologies, including stereotype threat, that impact older adults' uptake of technology and long-term health outcomes (Ivan and Cutler, 2021). Peine et al. (2014) note, “Older persons are assumed to follow what designers offer them, and it is therefore the tasks of designers to understand and meet the needs of older persons. This involves an uncomfortable framing of older technology users as passive recipients of technology” (p. 204). The ageist image of the passive older adult who receives technology as a victim of their environment conflicts with the recognised importance of older adults' making their places through “spatial agency,” or having control over their domestic space (Van Hees et al., 2021). In this context, exercising spatial agency would mean that older adults play an active role in decisions about the technology that enters their homes and how collected data is used. Research has found that older adults find agentive, creative ways to subvert monitoring systems in their homes (e.g., opening and closing doors and turning on faucets to mimic the surveilled behaviour) (Dalmer et al., 2022), but these actions do not appear in marketing campaigns, perhaps because it is the stereotypical images of docility, acceptance, and cooperation that sell technology.

AgeTech marketing discourse has been critiqued for its ageist constructs of ageing and older age. Advertisements rely on associations between older age and illness, decline, frailty, and forgetfulness. These associations in turn inform the definition of “needs” and justify the use of stereotypical representations of older people on marketing platforms (Neven and Peine, 2017; Peine and Neven, 2021). The problem of ageism in the media has been called “visual ageism” by Loos and Ivan (2018), which refers to underrepresentation and misrepresentation of older adults in the media, and “new visual ageism,” which refers to the “obsessive representations of older people in looking unrealistically young” (Ivan et al., 2020, p. 10). Acknowledgement of this has led to a call to push back against visual ageism in digital media content. Indeed, Einsend (2022) noted that the inclusion of older people in advertising has not been given enough attention by academics, calling for more research in this area.

In response to Einsend's call for action, this paper is interested in representations of older adults in AgeTech advertisements on the Internet, and how these may produce and perpetuate ageism in the marketing of sought-after smart home technologies. Taking into account that advertisements are multi-modal, comprising powerful combinations of visual and aural messaging, this paper will draw upon sound studies and media studies research, as well as Van Leeuwen's (1998, 1999, 2012) critical discourse analysis of music. The paper will explore how music, voice, and sound are used in three advertisements for ambient smart home monitoring designed to support ageing-in-place. The focus of the paper is on the relationship between the soundtracks and representations of ageist portrayals of older adults, the “technology as saviour” narrative, and the troubled boundaries of the ageing-in-place smart home.

## Representations of older age in AgeTech advertising

The market for technologies to support ageing-in-place is burgeoning alongside the neoliberal prerogatives of commercialisation, privatisation of care, and demands to embody the anti-ageing qua successful ageing lifestyle, particularly in the context of third-age older adults (Ylänne, 2015). Smart home ambient technologies are among the products. These are usually a collection of monitoring sensors and digital information-sharing devices that track daily activities, noting aberrations in routine. They assure risk mitigation through efficient and economical ambient monitoring devices that share collected data with older adults' grown children and care providers (Van Hoof et al., 2011). In contrast to the wearable emergency-call devices first introduced 50 years ago, today's ageing-in-place technology uses algorithms and “self-learning” intelligence to continuously monitor for aberrations in movement, behaviour, and vital signs (Urban, 2021). Marketing agendas simultaneously speak to the ethos of successful ageing (Rowe and Kahn, 2015), encouraging independence and self-responsibility for one's own wellbeing, and the filial obligation of adult children who are trying to balance full-time work schedules with “being there” for older adults in their lives. A pressing issue currently faced by marketers trying to catch up to ageing demographics around the world is the challenge of deciding when to market directly to older adults vs. third parties (e.g., family members, informal caregivers, and institutions) and how to go about creating honest portrayals that are neither negative, nor so positive that they reinforce age denial (Lamb and Gentry, 2013; Gentry and Mittelstaedt, 2017).

It follows that AgeTech marketing campaigns increasingly include representations of older adults. The target audience shifts from older adults as potential consumers to adult children and third-party caregivers. Technology always promises to solve impending disasters related to being older and living at home. Neven and Peine (2017) have articulated the ageing-and-innovation discourse in the pithy terms of a triple win and a triple sin. The triple win describes technology as a “saviour,” a saving grace, or the cost-effective solution for governments and individuals to ensure enhanced care for older adults who want to stay in their own homes (at least for those who can afford these technological upgrades in their homes), avoiding increased pressure on human labour. The triple sin includes the universal evaluation of technology as beneficial by constructing problems and devising solutions based on a necessary negative portrayal of ageing and “old” older adults. This ironically undermines uptake among older adults.

Researchers have noted that when we see representations of older adults in advertising, there is an ever-present risk of visual ageism that perpetuates distorted images of older adults, including reproducing stereotypes through “non-realistic, exaggerated, or distorted portraits of older people” (Loos and Ivan, 2018, p. 164). Other research shows that ageing is routinely problematised and pathologised, and that these processes are gendered, whereby older women are more likely to be negatively portrayed compared to older men, particularly in the context of resisting erasure, social exclusion, and loss of cultural capital (Coupland, 2007; Kenalemang, 2021). In the context of film and critical disability studies, there is a “triple bind” that faces older women that involves being cast as “female, disabled, (and) old” (Chivers, 2011, p. 57). Marketing is designed to play on underlying fears that consumers have been socialised to associate with ageing, as well as on our underlying social values. In the “cluttered mediascape” of advertisements, marketers use semiotic devices to grab audiences' attention and make their product memorable (First and Ramer-Biel, 2018). As Chivers noted about representations of older age in film, “old age appears to be, if not avoidable, at least manageable” (p. xix). Such an ethos appears in advertising, too. Gilleard and Higgs (2000) stated, “For now, at least, resisting age rather than ageism greases more palms, oils more deals, and turns more dollars” (p. 71). I would contend that it is now the *management* of age and the strategic use of ageism that greases, oils, and turns the AgeTech sales wheel.

Vermeer et al. (2019) were the first to explore online marketing for surveillance technology designed for both people living with dementia who were ageing-in-place, and for their care providers. Their analysis of marketing discourse across three countries (UK, Sweden, and Netherlands) found common emphasis on risk, safety, and security. The advertisements used a range of words across contexts from obvious surveillance language (e.g., “spy” and “camera”) to more disguised surveillance language (e.g., “sensor” and “discreet”), sometimes with tensions around uptake (e.g., “ethics,” and “consent”). Importantly, the messages were directed at families and care providers, rather than people living with dementia, who were dehumanised in their portrayal as a “problem to be managed” and categorised “in the same class as wallets, keys, young children, dogs and/or prisoners” (Vermeer et al., 2019, p. 27). Follow-up research by Vermeer et al. (2020) framed online advertisements for dementia-focused surveillance technologies as cultural texts that shaped audience perceptions about dementia and the impact that dementia has on others. This foundational research carefully analysed the content of YouTube advertisements, looking at the combination of visual representation and spoken language. While the authors mention music in the advertisements, there has not yet been an in-depth analysis of the soundtracks was not included. This paper intends to look at the combination of visual and verbal content in online marketing ads for AgeTech, but adds the use of sound (e.g., background music, on- and off-screen sound, and silence) to the analysis.

## Soundtracks and meaning-making in advertisements

The soundtrack of commercials is important in advertising as a means of connecting and communicating with the viewer. Sound gives the visual picture “body” as the ear makes the image visible (Chion, 1999; Elsaesser and Hagener, 2010). Chion (1999) argues that hearing takes sensory precedence over vision because of its ability to give substance and depth to the flat image on the screen. “Sound can hover on the border between meaning and non-sense, threatening to fall into the meaninglessness of babbling and muttering” (Elsaesser and Hagener, 2010, p. 137). It is this precarity that is powerful, imploring audiences to participate in the sense-making, the semiotic work of making meaning out of the performance on-screen. Particularly in the context of a soundtrack made up of music, silence, speech, and sound effects, there is a great deal of opportunity to make meaning in advertisements.

Music has been said to dominate commercials as a catalyst of advertising (Craton and Lantos, 2011). While there is debate about whether or not the presence of music always enhances the viewer's perception of a product (coined the “music as garnish assumption”), there is clear evidence that music is a tool that impacts viewers' emotions, cognition, and interpretation of the brand's message (Craton and Lantos, 2011). The selection of advertising music has the power to attract audiences' attention (e.g., if the music is novel, interesting, or surprising), enhance the memory of the ad's message and the brand as unique. Music also evokes emotions and sets the mood in commercials which influences audience perception (e.g., setting the ad's pace, triggering positive or negative emotional associations from past memories). There is a long history of the emotional power of music in commercials for popular brands, such as Coca-Cola, McDonalds, and Apple (Graakjær, 2015; Sánchez-Porras and Rodrigo, 2017). An important consideration for advertisers is the “perceived music-message fit” referring to “the consumer's judgment of how well the music suits the message, with high fit leading to a more favourable attitude” (e.g., upbeat music for a “fun” entertainment product, relaxing music for pain relievers and beer) (Craton and Lantos, 2011, p. 402). It stands to reason that the perception of fit among audiences relies on common social beliefs and understandings about the products and people represented in the advertisement. This can be particularly hazardous for reproducing ageist messaging.

Extending this point, background music in film and in advertising signals or “activates” schemas associated with extra-musical meaning (Boltz, 2001; Herget and Bötzl, 2021). As examples, background music can impact audience perception of credibility in advertising, and musical stereotypes align with gender stereotypes (e.g., masculinity is symbolised by electric guitars and rock music, while femininity is associated with violins, flutes, and romantic classical music) (Herget and Bötzl, 2021). According to Cheu (2007), the female voice is often perceived as symbolic of disorder, weakness, and unpredictability, while the male voice suggests order, authority, and structure. That which is perceived as feminine lacks rhythm and resists conforming to a fixed structure (Graham, 2016).

Sound studies scholars have looked at the impact of music on audiences' perceptions of the characters' emotions, relationships with other characters, and functions in the scene. Scholars have also examined the impact of music on audiences' interpretations of the film's narrative (Tan et al., 2007). Music's effect on the interpretation of visual content is explained by the idea that schemas are activated and guide viewers to focus attention on parts of the scene through temporal, mood, and semantic congruence between what is seen and what is heard. Music is particularly powerful when there is forward priming (music is heard before the character is seen) and with concurrent music (accompanying the character when they are seen). The “affective priming” leads audiences to interpret emotion on a character's face or in their physical posture in a particular way. Research has found that emotions like, anger, fear, sadness, and happiness are most accurately recognised through music. Film theorists continue to debate the impact of the “scene of empathy” (where the narrative slows to focus viewers' attention on the character's face) and the significance of music as a device to support the directing of attention to the character's face and emotional state (Tan et al., 2007).

## Methodology

Between September 2021 and March 2022, the author collected data through an online search for AgeTech advertisement videos for ageing-in-place technologies that were posted online between the years 2015 and 2022. Similar to semiotic analysis of AgeTech YouTube videos by Vermeer et al. (2020), the author used the video search function on the Google search engine to locate videos on YouTube. The search targeted specific AgeTech companies that had been identified in a previous research project about design of technologies for ageing-in-place among prominent companies that market ambient monitoring systems for older adults. The search string thus included the company name, “monitoring,” “ageing in place,” and “home.” A total of 15 concept videos from AgeTech companies were included in the data set for analysis. Videos were included if they had audible background music, voice components, sound effects and/or auxiliary sounds. For the purpose of the analysis and presentation for this paper, three illustrative videos were chosen for in-depth exploration and discussion. The author was the only person to select and analyse the advertisements.

### Analytic framework

Critical discourse scholar Van Leeuwen (1998, 1999, 2012) has contributed to models of multimodal discourse analysis that include critical analysis of musical discourse in mass media such as advertisements. He asserts that “musical signifiers, aspects of melody, harmony, musical structure, rhythm and timbre, can be convincingly linked to social meaning potentials in ways that help us analyse both the hegemonic ideological work and the counter-hegemonic work of music in society…” (p. 320). Sound, he says, communicates meaning based on our experience of it as sentient beings and how we relate what we hear to a given social context. “The semiotics of sound concerns itself with describing what you can “say” with *sound*, and how you can interpret the things other people “say with sound”” (Van Leeuwen, 1999, p. 4).

A methodological challenge of translating music, sound, and voice into text stands against historical tensions between those who categorise music as autonomous, abstract, and independent of social meaning and those who understand music to be a socially contingent text or discourse (Tagg, 2013). Siding with the latter, Van Leeuwen (1998, 1999, 2012) navigates the methodological challenge by outlining a set of tools for multimodal analysis. These tools are organised into audible musical systems (i.e., melody, harmony, rhythm, and timbre) that can be codified with (albeit imperfect) adjectives and descriptive language as what is heard is mapped social meanings and sociomusical references. For Van Leeuwen, musical texts are unique because they “create meaning simultaneously in different semiotic modes” (1998, p. 27). This is a powerful approach to analysing the AgeTech concept videos because what is simultaneously heard and seen by the viewer can be analysed in terms of social theory and sociological analyses of the given social context (i.e., the neoliberal approach to care for older adults using ambient home monitoring technologies with all the accompanying ethical tensions). Following Tagg (2013), it is worth noting here that the translation of music, sound, and voice into text relies on a shared vocabulary of musical sounds and signs, as well as sociocultural norms that inform the interpretation of the sounds. The analysis of the music, sound, and voice in the concept videos is based on the Western tonal system (the systemic-functional semiotics) defined by Van Leeuwen, binding the work within its given cultural milieu. The following section will detail Van Leeuwen's analytic categories that enable the translations of that which is heard into analysable text.

Drawing across the work of Van Leeuwen (1998, 1999, 2012) and Machin and Van Leeuwen (2016), the following analytic categories can be discerned: perspective, musical time, melody and sound acts, major and minor modality, and vocal quality and timbre.

#### Perspective

Musical perspective is used representationally to focus the listener's attention on different aspects of the event. While images represent social distance through the size of frame and perspective, sound represents social distance by creating relations of formality between the video and the viewer. Van Leeuwen (1999) explains how each perspective is accomplished: “intimacy (the very close shot, the whispered voice), informality (the close or medium close shot, the relaxed casual voice), formality (the medium long or long shot, the louder, higher and tenser voice which “projects” the message)” (p. 15). Additionally, sound can be placed in the musical foreground (“the figure”), front and centre in the listener's attention. Sound may also be positioned either in the middle ground as the social setting, which is heard, but not listened to, or in the background (or “field”) as the more distant social setting. Van Leeuwen (1999) notes that microphones and amplification technology have blurred the distinction between private and public because a whisper captured by a microphone can easily by amplified and projected across great distances.

#### Musical time

Time in music “enacts and celebrates the timing of social interaction” (Van Leeuwen, 2012, p. 324). Musical time regulates activities and represents characteristics of that time to the viewer who entrains to the rhythm and emotionally identifies with the presented characteristics (Van Leeuwen, 1999). Drawing upon the social history of the clock, industrialisation, and controlled labour, Van Leeuwen connects the discipline of measured time (and tempo) to disciplinary institutions, such as schools, hospitals, and prisons. Conversely, unmeasured time lacks a feeling of steady beat signifying the eternal, the divine, and that which is “not human.” Such classifications of musical time make up “sound scripts” where rhythmic changes draw “musical pictures(s)” of the content itself (Van Leeuwen, 1999, p. 64–65).

#### Melody and sound acts

We do things with sound through “sound acts” just as we do things with words in speech acts (Van Leeuwen, 1999). The dividing line is thin, says Van Leeuwen (1999), between speech, music and other sounds. Thus, with any of these, we can “announce our presence, hail, warn, call or help, lull to sleep, comfort, and much more” (p. 92). While social context ultimately gives music and sound its meaning, values and identities can be represented through sound acts created by melodic direction and pitch range (the emotional extension of sound acts), pitch level (representing dominance and diminution), and character of music and voice (depicting actions and qualities of people, places, and things). For example, higher *pitch* and *ascending melodic lines* are associated with energy, outgoingness, and brightness. Lower *pitch* is associated with lower energy and *descending melodic lines* are felt as more passive, incoming, and inward-looking. Further, the intervallic movement of the melodic line may be associated with either masculinity and heroism when steps are large, or femininity and sentimentality when steps are small and chromatic. A voice that is higher and sharper may increase listeners' feeling of tension, while a lower voice may be associated with a relaxed mood.

#### Major and minor modalities

The major mode is positioned as the western norm. It is associated with progress, achievement, and positive values of industrialisation, science, and industry. The minor modality is associated with standing in the way of progress, depressing the major modality. As mentioned earlier, major and minor modalities are associated with emotion. Van Leeuwen (1999) explains, “In the West, we have of course “privatized” the discourse of music, and speak of major and minor in terms of “mood,” with major as “happy” and minor as “sad,” but in fact music fuses ideological meaning an emotion, and it is precisely therein that its power lies” (p. 325).

#### Vocal quality and timbre

Van Leeuwen (1999) wrote, “Sound never just “expresses” or “represents,” it always also, and at the same time, affects us…There is always both the social and the personal, both meaning and pleasure—or displeasure. The difference lies in how we *value* the social and the personal, or meaning and pleasure, and in the degree to which we acknowledge their unavoidable interconnections” (p. 128). Voices are a combination of features (e.g., soft/loud, high/low, tense/lax, and rough/smooth) whose presentation and meaning are graded. Qualities of voice, such as breathiness and softness are associated with intimacy, confidentiality, and that which is private and feminine. Louder, amplified sounds are associated with assertiveness and the public realm. Van Leeuwen also considers how sounds are either plain and unwavering, or vibrating and trembling, and the association of these sound qualities to audiences' emotions.

## Results and analysis

This section details the soundscape of three smart home monitoring technologies marketed to support ageing-in-place. The description of each video details the audio soundtrack of each advertisement, including the background music, voices, and sound effects, as well as the visual material that is accompanied by the soundtrack material.

### Video 1: Essence Care@Home video (2020)

Essence SmartCare is part of Essence Group, an American company that provides smart home technology to support ageing-in-place through home care monitoring indoors and outdoors. The video for their Essence Care@Home product is 1 min and 23 s was available on their YouTube Channel (Essence, 2020).

The video opens with soft minor chord progression moving at a *grave* tempo of 30 beats per minute (bpm) in a measured and steady time. Sombre chords accompany the next two scenes. Each chord sounds and decays into nothingness, emphasising the image of the older woman who is looking lonely, exhausted, and depressed. Following Van Leeuwen (1999), the minor modality depresses the major modality, standing in the way of positive values of achievement and progress, and signalling privatised emotions of sadness. The minor chords here parallel the concerned voice-over scripts of the adult children The video continues to alternate between two older adult protagonists: one female and one male.

The opening scene is of an adult daughter driving her car down an urban street in the early morning, looking up at the façade of an apartment building. The viewer is positioned in the back passenger seat, looking forward at the side of the adult daughter calling her mom on speaker phone:

“Hey Mom!”

The video continues against the dirge-like chords, showing the mother in her apartment alone, sitting at the kitchen table in a red fleece snowflake-patterned bathrobe. Her eyes are cast downward, with one hand placed on the side of the coffee mug (no hand on the handle). The daughter's voice-over says:

“I love my mother, but I never thought it would be such a challenge to take care of her as she got older, and it has become even harder since my father died. I talk to her every morning and I would like to see her more, but I can't be in two places at once.”

The voice continues over video of the mother looking like she is having trouble breathing, her face drawn as she moves into a chair in her living room while holding her head. We see and hear her loud sigh, looking exhausted after she sits in the chair. Kress and Van Leeuwen (2020) characterise this as an offer gaze, literally presenting herself as an object to the audience, but conveying powerlessness and denial of personhood through her passive pose and sigh connoting a life that is weathered and worn-out.

The same minor chords continue in the second scene. The images switch between an older adult male in his bathroom putting on his bathrobe, and a younger businessman (his son) on a cell phone in a work setting. Against the slow pace of the minor chords that convey concern, the adult son's voice-over says:

“Dad was always there for me. Now I want to be there for him, too. But every time I leave his house, I wonder “what if something happens to him and he won't be able to reach the phone.””

Just as the son says, “what if something happens…,” the viewer sees the older man's feet slip on the wet bathroom floor and we hear him call out “Whoa!” as he falls. We hear the sound effect of a fall (which sounds like a crunch) and then the man calls out again, “Ahh!” as if in pain. The video shows him lying on his back between the toilet and the bathtub, his eyes wincing, arms by his side, groaning in pain, “Oh, oh…”

Suddenly, the high-pitched sound of the Essence Care device is heard with alarm-like ringing. The video moves to the image of the device on the bathroom wall with superimposed sound symbols emanating from it. The sombre chords switch to somewhat louder piano music played at a faster-paced *moderato* tempo of 120 bpm. The melodic line is dance-like, featuring a busy, quick-moving ascending three-note motif that includes major 3rd intervals. The melodic line repeats against the same set of minor chords. The music creates a sense of urgency, immediacy, and attention. Van Leeuwen (1999) noted that while small chromatic (stepwise) intervals mean sentimentality in Western music, the larger intervals connote heroism.

The background music continues as a male voice-over from the company says:

“This is why we are here. We are your eyes and your ears, 24 h a day, always on the watch.”

The scene cuts again to an image of the mother at home in her chair and the white monitoring device beeping behind her. The beeping and voice surprise her and she looks up at the ceiling seemingly unsure where the voice is coming from:

“Hello, Mrs. Andrews, this is Adam from Care at Home. We would like to send over a doctor to make sure you are well.” The video cuts between the mother and a male call agent speaking to her through his headset.

Against the similar music, this time with the chords doubling in speed, but still structured as minor chords beneath a melodic motif with major intervals, the viewer sees a female call agent say to the older man who is holding his side in pain on the bathroom floor:

“Mr. Jameson, this is Kate from Care at Home. Don't worry, we're sending a team to help you.”

The video shows a still image of the device and its log of activity with a red ringing alarm bell on a tablet screen. The daughter's voice-over continues as the viewer sees the mother being taken care of by a female doctor in her home:

“One of the best decisions I ever made was to instal Care at Home at my mother's home. Now I have peace of mind because I know Care at Home will be there to help in my mother's time of need.”

As the image switches from the fallen father to the adult son and the device itself, we hear the voice of a company representative say, “An intelligent homecare solution…” The background music pauses on a single chord and suspends it in unmeasured time before continuing, “…enabling families to be confident that…,” the music fades to silence as the voice says, “their dear ones are cared for…” The voice pauses and with a synthesised *whoosh* sound, the voice continues over an image of the technology, saying, “…while enabling seniors to maintain an independent lifestyle.”

In this final segment of the advertisement, the music reverts back to the *moderato* tempo of upbeat music heard before, but this time with synthesised strings playing instead of piano. According to film study theorists (Boltz, 2001; Herget and Bötzl, 2021), background music can have extra-musical meanings by activating schemas. One of the musical stereotypes associated with gender is that femininity is associated with the sound of string instruments (e.g., violins) and the sentimentality associated with the vibrato in the sound (Van Leeuwen, 1999). The strings accompany visuals of the adult son at home checking his father's data log on his smart phone, and of the mother and daughter smiling and hugging at the mother's kitchen table. The adult son's voice-over says, “with Care at Home, I worry less.” The music ends and the company representative's voice-over states the slogan, “Essence. Better life made possible.”

### Video 2: Vayyar imaging (2018)

Vayyar is an Israeli company that offers ambient home monitoring technology for ageing-in-place. The video was 1 min and 34 s and was available on the BusinessWire YouTube channel (BusinessWire, 2018).

The video begins with an energetic adult man outside on a city sidewalk beneath trees. He is filming himself using his phone, one arm extended upward holding the camera on selfie mode, and the other hand holding up the Vayyar sensors. The background music is present, but not louder than his voice. Playing at an *andante* tempo of 88 bpm, the music is upbeat, positive, and cheery. The high-pitched playful notes of the piano against the casual strumming of a ukulele create a carefree, uncomplicated, music box-like atmosphere of simplicity and ease. This melody is in the middle ground of musical perspective, providing the social setting and context (Van Leeuwen, 1999).

The fellow says, “I got these two sensors at Vayyar. I'm going to Grandma's house (gesturing with his head to beyond the sidewalk), we'll see what happens.”

The music continues at the same volume, accompanying a scene where the grandson is installing the sensors on the wall with his grandmother.

Grandson: “Snaps right into the wall.”

Grandmother: “Is this thing filming me?”

Grandson: “No, doesn't use a camera. It even works when you cover it up (lifting a painting and placing it on the wall over the sensor).”

Grandmother: “Yes, but what does it actually do?”

The same background music continues at a louder volume accompanying an accelerated video of the grandmother zipping around her apartment kitchen preparing food, lying in bed while putting on her glasses to read her sleep score on her smart phone, playing with her grandson and great-granddaughter, and reclined on her couch reading a tablet. Over the video, written (unspoken) bullet points that answer the grandmother's questions about the product: “lights, a/c, appliances, monitors sleep and improves sleep (no wearable), monitors your activity level (showing steps being recorded on the screen), even can direct your Wi-Fi towards you.”

Still in an accelerated video mode, the grandmother is filmed getting up from the couch and going off-camera with her tablet. The background music fades and the sound of running water increases in volume. There is a moment of silence and then the sound of a heavy fall (like a falling bag of flour). The tap of a tom drum (thump) and the rapid prestissimo ticking of a timer (282 bpm) is joined by a high-pitched beeping from the smart phone seen on the screen: a hand holds a smart phone. The phone screen is bright red. The alert reads “Fall detected” with a shower icon and a twitching red alarm bell graphic with sound waves around it. The bottom of the phone screen says, “Call 911.” The viewer sees two more cellphones held in hands at different angles, suggesting that three different family members have now received the fall alert.

The drum sounds again, the alarm beeping stops, but the rapid ticking continues as the grandson opens the door, and rushes to the bathroom off-screen. The viewer sees his whole body from far away, signifying greater personal distance. Even his voice sounds soft and distant when we hear him call, “Grandma!” as he runs to help her. The viewer hears the reassuring voice of the grandmother say, “I'm fine. I'm okay.” Her voice sounds quite close to the viewer, at a much closer distance than the far-off voice of the grandson.

The screen fades to white and the upbeat music resumes. The grandmother is seen from behind at her kitchen island, video chatting on her laptop with a young woman on the screen. The app on her laptop shows a figure approaching the door, and she opens her apartment door to her grandson holding flowers and a gift bag. The background music volume decreases as she greets him with a warm smile and a strong voice saying, “Hi! What lovely flowers!” As he starts to hand her the gift bag and flowers, he says, “Grammy, I brought you a bunch of stuff…um, also, I've got to get the sensors back.” She frowns and closes the door as the upbeat music fades back in and resumes at full volume.

### Video 3: SofiHub Home@JB Hi-Fi (2020)

SofiHub is created by an Australian company that provides ambient home monitoring and digital reporting through smart technology. The advertisement being analysed is 2 min and 26 s long and was available on the JB Hi-Fi YouTube channel (JB Hi-Fi Official, 2020).

The video begins with an image of the SofiHub device accompanied by upbeat piano music in the background at a very low volume. It is a major key and playing at a *moderato* tempo of 110 bpm. The video shows SofiHub on a table in a close-up shot, lighting up as it says in an automated, lower-register female voice, “Good morning. Today you have a doctor's appointment at mid-day.”

The background music continues at the same level, underneath the speaking voice of the SofiHub representative who takes over the script. The background music is thus located at a more distant social setting in what Van Leeuwen (1999) calls the “field,” as the SofiHub representative takes over the narrative, explaining the features of SofiHub while standing in a kitchen with the SofiHub device on the counter behind her. She explains:

“SofiHub Home is an ambient assisted living technology, designed to promote the wellbeing and independence of the ageing, as well as individuals with levels of disability. It uses smart technology combined with spoken reminders to encourage positive living and adherence to health-related routines. Through intelligent monitoring of movement using motion sensors placed around the home [as the video switches to panning the perfectly made, white linen bedroom and spotless white tiled bathroom], it also detects behavioural change (a male arm is shown placing a sensor in a doorway] and automatically initiates alerts to the appropriate contacts. All of this is achieved in a non-invasive manner [showing an older woman in a sunhat outside pruning an ornamental tree], ensuring an individual's right to privacy [the video pans vertically upward, showing an older man in a white undershirt sitting on his bed with his hands resting on a cane with the white curtains drawn]”.

The script continues without pauses as the video scene switches to a woman in her 30 s transferring herself from her wheelchair to a couch in her apartment to visit with her cat. The representative's voice over says, “The device has peace of mind alerts which notifies…” As the woman makes contact with the seat of the couch, the volume of the background piano music is increased and a quick drum track takes over from the piano's melodic line. The tune is still present, but the listener's ear is drawn to the drum's quick metronomic pulse and novel energy. At the point of contact, the voice-over script continues, “…carers and family members *via* text message when anomalies are triggered in three key routines…” The video switches back to the representative, with a high-pitched “ding” sound effect marking a blue text bubble popping up at the top left of the screen reading “ANOMALY TRIGGERED: Trevor is late to rise. Would you like to cheque up on him?” The script continues, “Late to bed, late to rise, and long duration in the bathroom.”

As the script continues about the device's capabilities paired with images of a picturesque clutter-free home, the drum track fades out of the music. The music returns to a more relaxed mood with light electric guitar lines taking over from the piano. For Van Leeuwen (1999), ascending melodic motion is associated with energy and brightness, giving a hopeful tone to the end of the advertisement.

The music alternates back to the variation with the drum track and slightly louder—but still gentle—electric guitar riffs. This more excited variation of the music accompanies the representative explaining, “It allows carers to send text to voice messages to our device remotely *via* our online portal or an iOS or android smart phone app.” The video cuts to an older adult woman and a daughter sitting side by side on a couch smiling and laughing together with the daughter's arm around the mother's shoulders. The video then shows the online interface through which one can write a text message that gets delivered to the SofiHub device. The video continues with a clip of SofiHub speaking the message that was typed in the scene before, “Hi mom, don't forget that I'm coming to see you today at 6 pm.”

The final scene of the video shows the representative saying, “SofiHub advanced adaptive care technology gives people of all ages and abilities the freedom and confidence to live independently.”

## Discussion

The analysis of the three advertisements showed that soundtracks can be just as carefully scripted as dialogue. Moreover, the way music, voice, and sound (i.e., sound effects and auxiliary sounds) are deployed reinforces existing stereotypes and problematic portrayals of older adults and ageing. Examples of the interplay of audio and visual material with ageist discourse in the selected AgeTech advertisements will be discussed below.

### Sounding ageism: Soundtracks and stereotypical portrayals of older adults

One of the main problems with representations of older adults in advertising, and in AgeTech advertising in particular, is visual ageism (Loos and Ivan, 2018). As noted earlier in the paper, representations of older adults are more than likely overly negative because the focus on risk, the threat of the home environment, safety, and security is how companies sell their products (Vermeer et al., 2019, 2020). Representations of older adults in advertising can also be unrealistically positive (“new visual ageism”) which encourages age denial and reinforces ageing as problematic and pathological (Ivan et al., 2020). This tracks with what Gilleard and Higgs (2010, 2022) pointed to as a duality between utopian and dystopian futures of ageing, or the third-age imaginary where technology is adopted to increase enjoyment and pleasure in life, vs. the fourth-age imaginary (or “real” old age) where technology is used as a necessity to manage corporeal decline. It is important to turn our attention to how visual and acoustic ageism work together to represent older adults and their possible futures.

Negative stereotypes of older adults were common to the AgeTech advertiseents. The vision of the older adult woman in the Essence Care video most profoundly reflected the dystopian, fourth-age imaginary of dependence, impairment, and lack of agency, not only visually, but also acoustically. As discussed above, the background music set the scene for the viewer to perceive passivity, lethargy, and deterioration. The acoustic dimension of the negative fourth-age imaginary is characterised by slow-moving, descending and decaying musical lines that are simultaneously passive (un-agentic, in Gilleard and Higgs' terms), and ominous. Just as Neven and Peine (2017) state that the ageing-and-innovation discourse stigmatises older people as old, so, too, can the musical discourse of AgeTech advertisements.

Advertising researchers note that music creates mood through both pacing and the triggering of emotional associations from past memories (Craton and Lantos, 2011). Affective priming in particular can impact audiences' perception of characters before they appear on-screen. By design, this is what takes place at the beginning of the Essence Care and Vayyar ads. Following Van Leeuwen (1999), the minor modality depresses the major modality, standing in the way of positive values of achievement and progress, and signalling privatised emotions of sadness. The Essence Care ad portrays the older adult woman more negatively than the older adult man by focusing attention on her face in a “scene of empathy” (Tan et al., 2007) and her audible exhausted sigh. Together, these representations reinforce the association between ageing and decline. Interestingly, it has been noted that these overly negative portrayals of the much-dreaded fourth age alienate older adults from technology because they identify technologies as being for “old people,” a social category with which they do not identify. Thus, both visually and acoustically (which this paper highlights), barriers to technology engagement are created through negative portrayals of older adults.

Following the binary pattern of dystopian-utopian imagined futures, the Vayyar advertisement provides an example of a utopian, agentic, third-age future with smart home technology. The background music is carefree, uncomplicated, almost toy-like in its simplicity and ease, setting a scene of leisure and play. Craton and Lantos (2011) note that upbeat music can symbolise fun entertainment products in advertising soundtracks. Framing the technology as an “entertainment technology” may help to bypass the tensions associated with surveillance technology. The music sets the stage for the audience to perceive the older woman as an agentic, third-age consumer who adopts technology to make life more enjoyable and less onerous (Gilleard and Higgs, 2022). This fits with common stereotypes of the “Golden Ager,” the “Perfect Grandparent,” and “the Productive Golden Ager” who is portrayed as full of “zest” and living in intergenerational harmony (Ylänne, 2015, p. 371).

A most remarkable feature of Vayyar's positive portrayal of the older adult woman is the strategic use of accelerated video and increased volume. As the video fast-forwards through the older woman's daily activities, the technology is shown working quickly to track her movements. This reflects an exaggerated version of the agentic third-ager whose fitness and power (according to Gilleard and Higgs, 2022), contrast with the overwhelming corporeality of the fourth-age. The grandmother is made superhuman, pushing towards the edges of the “*humachine* imaginaries” (noted by Gilleard and Higgs, 2022) and the horizon of possible technological enhancements for older adults. This is perhaps verging on a representation of the “technogenarian” whose embodied experience of technology is agentic, actively shaping, adapting, and even hacking gerontechnologies (Bishof and Jarke, 2021, p. 207).

Just as there are risks associated with negative stereotypical portrayals of older adults, there are also risks of overly positive portrayals. Namely, there is the danger of encouraging “age denial” which reinforces overly youthful portrayals of what ageing-in-place with technology looks like. These portrayals of intergenerational relationships around technology adoption and in-home surveillance are also problematic because they gloss over ethical tensions and power asymmetries (Berridge, 2016, 2017; Berridge and Westle, 2019; Pirzada et al., 2022) in favour of a dream of agelessness (Gilleard and Higgs, 2022). Thus, in comparing these AgeTech advertisements, there is a binary acoustic representation of dystopian and utopian futures and, as Gilleard and Higgs (2022) point to, a real gap between lived experience and imagined futures with smart home technologies. The next section of the paper will take up the role of music, voice, and sound in constructing the narrative arc of the advertisement.

### Rescue soundtracks: Acoustic constructions of the “technology as saviour” narrative

An important concern about technology and ageism is the portrayal of technology as a “saviour,” or the efficient and effective solution to ensuring safety and security for older adults wishing to age in place in an otherwise threatening home environment (Neven and Peine, 2017). This follows the grand narrative or gospel of *tech solutionism*, a concept that became known through Evgeny Morozov's book, *To Save Everything, Click Here* (2013). This discourse positions technology as the “fix” for complex social issues (Brevini, 2021; Byrum and Benjamin, 2022). Brevini (2021) notes that this kind of myth-building is commonplace in tech solutionism, observing that each new wave of technology first brings declarations of an end, or crisis, which is quickly forgotten with each subsequent wave. Indeed, the “ageing enterprise” profits by constructing ageing as a crisis it can solve (Neven and Peine, 2017; Carver and Mackinnon, 2020).

In the AgeTech advertisements, tech solutionism is articulated through the narrative arc that always includes an accident (usually a fall) or health crisis for the older adult at home, amplifying vulnerabilities (as surveillance capitalism does) before offering a solution (Carver and Mackinnon, 2020). In each case, the smart home technology came to the rescue. The interesting feature of these advertisements is the important role of music, sound, and voice (and silence, too) for constructing the crisis for the viewer. As described in the previous section, the crisis in the Vayyar advertisement is entirely heard. The grandmother disappears off-screen, and the viewer hears running water for a short time before the background music fades to silence and a sudden “thump” of the tom drum (like a body falling) interrupts the flow. This is the crisis moment. Immediately afterwards, the urgency of the situation is scripted by the rapid ticking, and is paired with high-pitched electronic beeping on three different smart phones held by caregivers with whom information is shared. Here, technology knows about the crisis before people do and comes to the rescue with an immediate alert. The acoustic perspective at the end of this crisis also positions technology in the foreground and the human (the grandson) in the background, almost late to the scene. As the grandson runs into the grandmother's apartment, the volume of his voice is lower, distant, and further from the viewer. It acoustically emphasises that technology is on the scene first.

Brevini (2021) notes that tech solutionism involves the myth that technology will surpass and outperform human capabilities. Indeed, the passive monitoring system is the first to know and to send alerts about the crisis. An even more poignant acoustical scripting of technology as saviour is evident in the Essence Care advertisement. Following two health crises (a fall and a potential heart attack), the minor *grave* piano chords are immediately replaced by sudden high-pitched sounds, and alarm-like ringing of the device and faster background music. The sudden quickening of time suggests urgency. Technology makes its appearance as the hero through wider intervallic steps in the music and literally a “whoosh” sound effect, as if it is flying in to save the day. The disembodied voice-over seems to come from above, as an omniscient protective saviour in the storey. Here, technology again knew first and connected the older adults with help before a family member could, thus maintaining the myth of technology transforming our world and exceeding human capabilities.

Each advertisement concludes with a “happy ending.” The older adult has been rescued by technology and reunited with loving family by their side. As Neven and Peine (2017) explain, ageing-and-innovation discourse for older people (particularly a “dear old lady”) is positioned as inherently good (p. 7). There are “only winners” when innovation can save us from the ageing apocalypse (Neven and Peine, 2017, p. 7). Indeed, the authors note that moral high ground is created because few would disagree with a technology that promises to allow older adults to age safely in their homes. Acoustically, there are subtle ways to communicate the taken-for-granted nature of technology as a beneficent, non-invasive enhancement to everyday life.

The subtle changes in the background music of the SofiHub advertisement are an example of how music reinforces the normalisation of surveillance technology. There is no dramatic “crisis” in this advertisement, but there is still a construction of a need that key routine behaviours (late to bed, late to rise, and long duration in the bathroom) require monitoring and reporting to care providers. The inclusion of the more energetic drum track primes the viewer to perceive the technology as supporting successful, active everyday life and watching out for any sign of decline. According to Van Leeuwen (1999), ascending melodic motion is associated with energy and brightness, suggesting that the inclusion of electric guitar slides provides a happy and hopeful ending to the advertisement, ensured by the use of technology. In this case, everyday life with technology is pictured as good—the older woman gardens safely, the older man maintains his privacy. Interestingly, the change to the music occurs when a younger adult woman successfully transfers from her wheelchair to her couch, accompanied by a well-timed shift in the music to a louder, more energetic mood through the addition of a drum track. The use of a younger adult may be an effort to reduce stigmatisation of the device as a tool for “old people” (a barrier to uptake discussed above). The technology is there to save the day for everyone at home and the blending of the soundtrack into the background may strategically reinforce the message that technology is non-invasive and can simply become part of everyday life.

As music, sound, and voice are used to construct the saviour discourse in the techno solution milieu, there is a risk that viewers will uncritically accept monitoring technologies. Recalling Neven and Peine's (2017) observation about technological solutions and construction of the moral high ground, the acoustics of AgeTech advertisements reinforce the perception that technology is for common good, making humanity “flourish” by enhancing personal and societal wellbeing (Brevini, 2021). Carver and Mackinnon (2020) note that technological fixes can infringe on older adults' rights to independence and self-determination. Technology comes to be seen as a minor threat compared to the perceived risks and threats to older adults' safety and security within the home. Indeed, technology becomes the saviour that is “necessary, caring, and even freeing,” maintaining the “false dichotomy between safety and surveillance” and obfuscating concerns about privacy and autonomy (Carver and Mackinnon, 2020, p. 217). In the following section, the role of music, sound, and voice in the construction of private and public space will be discussed.

### When the walls talk: Troubled boundaries of the ageing-in-place smart home

A final issue related to the marketing and uptake of technology for ageing-in-place is the way in which “home” and embodied lifeworlds shape and are shaped by technologies (Peine et al., 2014). Urban (2021) noted that the boundaries between private and public domains become troubled in the smart home. The ideal of home as synonymous with autonomy and privacy can no longer be maintained with the continuous channels of information flowing between the private and public spaces. The continuity of the domestic, private sphere is interrupted, pierced, and punctuated by technology, becoming a “securityscape” (Low and Maguire, 2019). In a securityscape, security is domesticated and bodies are made visible and intelligible through technological monitoring and digital translations of information. Everyday experience is one of living security and experiencing threat and protection within the home as a multiscalar world (Low and Maguire, 2019).

The soundtracks of the advertisements become what Urban (2021) called the “performative process of materialisation” (p. 65). Van Leeuwen (1999) suggests that soft and loud sounds are associated with different degrees of social distance. Soft sounds are associated with close, intimate, private personal distance, while loud sounds are associated with that which is further away and more public. As noted above, SofiHub's background music is not particularly remarkable, but it is precisely its continuity without interruption in everyday activities that holds the perimeter of the private space. This is not complete, however, because the closing scene shows a text message from an adult child being translated through technology and delivered into the home through the electronic, impersonal tone of the SofiHub voice. Of the three advertisements, this gives the audience the greatest sense of separation between private and public domains where home-life is uninterrupted (as represented acoustically).

Vayyar's advertisement more clearly troubles the boundary between private and public domain through music, sound, and voice. The foregrounding of loud, peppy music that is a genre unlikely to be heard in the home immediately superimposes the public onto the private. Additionally, the imposition of instruments and sounds that typically do not belong to the private domain (e.g., the tom drum, the rapidly ticking timer, the high-pitched alarms beeping on three different smart phones beyond the pictured private home) press the outside into the private space. Towards the end of the advertisement, there is an inversion of public and private. The grandson's voice is soft when he calls out upon entering the grandmother's space, but the grandmother's voice is loud and close to the viewer, as if it was in the public space of the viewer. Visually, the final scene where the grandmother shuts her apartment door (the physical border zone between private and public space) on her grandson, keeping the technology inside with her, signals an integration of the public into the private.

Finally, Essence Care represents the clearest interjection of public into private domains. Whereas, SofiHub translated a personal message into a generic automated voice with which one could perhaps become familiar over time, Essence Care pierces the domestic space with the voice of an unknown caller from the company who goes only by their first name (e.g., “Adam” or “Kate”). The voices are loud and strident as they are projected through the technology, calling from far beyond the home, the sound symbolising the formal or public distance. In the scene of the older woman hearing the call, she looks surprised, casting her eyes to the ceiling in search of the source of the disembodied voice. The utterances and sighs of the older adults are softer, connoting private, intimate space, but the voices of the adult children and the company representatives outside the home are much louder, connoting the public domain.

In the case of both Essence Care and Vayyar, the home space (particularly the bathroom) is constructed as a threat to older adults. Parallel to Berridge (2017) finding that the presence of sensors in the bathroom can increase the risk of falling because a person feels surveilled and rushed, the soundtracks of the advertisements reinforce the bathroom as a place of risk and monitoring in the minds of viewers. The soundtracks grab the audience's attention and reinforce anxieties and hyper-awareness about one's vulnerabilities at home (Urban, 2021). Adding to the discussion of the embodied experience of ageing and technology, the near-instantaneous event of a fall and its detection is the point at which the border between the private and public domains is most obviously transgressed in social relationships around technology. Interestingly, in terms of the subjective experience of falling, Katz (2015) has written that falling is the “intersection point between the inside and outside of ageing” (p. 165). Though it is beyond the scope of this paper, the soundtrack of crisis events, such as falls, in the context of AgeTech advertising deserves more focused attention.

Following Urban (2021), the entanglements of technology make the home a “contingent phenomenon,” performed at an ever-shifting nexus of institutionalisation and being at home (p. 65). While the troubling of public/private boundaries is a ubiquitous problem associated with home technologies (e.g., remotely controlled webcams and cell phones that listen in), the normalisation of these boundary violations in older adults' life worlds is important to discuss. In terms of materiality and phenomenology, it is important to continue to think about asymmetrical relationships of power, security, and risk, and about experiences of ageing-in-place.

## Future directions

This paper has explored the use of music, voice, and sound in smart home ambient monitoring technology advertisements designed to support ageing-in-place. The analysis focused on addressing key concerns about AgeTech design, including stereotypical portrayals of older adults in technology advertisements, uncritical reproductions of the “technology as saviour” narrative, and the construction of “home” in the smart home environment for older adults. The analysis of the videos identified ways in which background music, voice, and sound effects reinforced visual ageism (very negative portrayals) and new visual ageism (unrealistically positive portrayals) (Loos and Ivan, 2018; Ivan et al., 2020). Importantly, this paper emphasises that what is heard is more important than what is seen in advertisements. Thus, the paper takes the position that stereotypes in advertisements about ageing and older adults are as much acoustic as they are visual.

Further, music, sound and voice are powerful tools for creating a narrative arc in the advertisement. The soundtrack carries the storey from the opening scenes that set the social context and audience perception of ageing and older adults, to the moment of crisis, and finally to the point when technology saves the day and ensures a happy ending. The acoustic dimension is essential to convincing the audience that technology is the solution to their worries. The persuasive impact of music must not be underestimated, and further study into music and sound is needed to better understand how AgeTech marketing campaigns construct heroic tales of technology as the rescuer.

Finally, future work needs to look at how advertising soundtracks represent the ageing-in-place home. This paper has suggested that the ageing-in-place home is a securityscape, a multiscalar space where private and public domains are both imbricated upon one another and piercing one another's boundaries. The acoustic representation of crisis in advertising is impactful, grabbing the audience's attention and reinforcing the idea of the home as a place risk and threat. Additional work should look specifically at the acoustic representations of borders, both material and phenomenological, in the smart home designed for older adults.

AgeTech is a rapidly growing field and there is no sign of it slowing down as demand will increase in the coming years. Twinned with advancing AgeTech will be ageist discourses used in marketing campaigns. Music, voice, and sound are the catalysts in advertising, and their power to persuade and to shape perceptions should not be underestimated. When it comes to constructing and reproducing ageist discourses to sell AgeTech products, it is imperative that research use a critical multimodal lens to capture how acoustic and visual material are working together and independently. This paper has made strides in this direction, but more research is needed if we are to keep pace with technology and fight back against ageism in both AgeTech advertising and society writ large.

## Data availability statement

The original contributions presented in the study are included in the article/supplementary material, further inquiries can be directed to the corresponding author/s.

## Author contributions

The author confirms being the sole contributor of this work and has approved it for publication.

## Conflict of interest

The author declares that the research was conducted in the absence of any commercial or financial relationships that could be construed as a potential conflict of interest.

## Publisher's note

All claims expressed in this article are solely those of the authors and do not necessarily represent those of their affiliated organizations, or those of the publisher, the editors and the reviewers. Any product that may be evaluated in this article, or claim that may be made by its manufacturer, is not guaranteed or endorsed by the publisher.

## References

[B1] Berridge C. (2016). Breathing room in monitored space: The impact of passive monitoring technology on privacy in independent living. Gerontologist. 56, 807–816. 10.1093/geront/gnv034 26035900

[B2] Berridge C. (2017). Active subjects of passive monitoring: Responses to a passive monitoring system in low-income independent living. Ageing Soc. 37, 537–560. 10.1017/S0144686X15001269 28239211 PMC5321543

[B3] Berridge C. Westle T. F. (2019). Why older adults and their children disagree about in-home surveillance technology, sensors, and tracking. Gerontologist. 60, 926–934. 10.1093/geront/gnz068 31102442 PMC13377232

[B4] Bishof A. Jarke J. (2021). ‘Configuring the older adult: How age and ageing are re-configured in gerontechnology design,” in Socio-Gerontechnology: Interdisciplinary Critical Studies of Ageing and Technology, eds Peine A. Marshall B. L. Martin W. Neven L. (London: Routledge), 197–122. 10.4324/9780429278266-18

[B5] Boltz M. G. (2001). Musical soundtracks as a schematic influence on the cognitive processing of filmed events. Music Perception. 18, 427–454. 10.1525/mp.2001.18.4.427

[B6] Brevini B. (2021). Creating the technological saviour: discourses on AI in Europe and the legitimation of super capitalism, in AI for everyone?: Critical perspectives, eds Verdegem P. (London: University of Westminster Press), 145–159.

[B7] BusinessWire (2018). CES 2018: Vayyar Imaging Unveils New Smart Home Sensors [Video]. Available online at: https://www.youtube.com/watch?v=BLgOHEBkFdY (accessed July 31, 2022).

[B8] Byrum G. Benjamin R. (2022). Disrupting the Gospel of Tech Solutionism to Build Tech Justice. Stanford Social Innovation Review. 10.48558/9SEV-4D26

[B9] Carver L. F. Mackinnon D. (2020). Health applications of gerontechnology: Privacy, and surveillance: a scoping review. Surveill. Soc. 18, 216–230. 10.24908/ss.v18i2.13240

[B10] Cheu H. F. (2007). Cinematic Howling: Women's Films, Women's Film Theories. Vancouver, BC: UBC Press.

[B11] Chion M. (1999). The Voice in Cinema. New York, NY: Columbia University Press.

[B12] Chivers S. (2011). The Silvering Screen. Toronto: University of Toronto Press.

[B13] Coupland J. (2007). Gendered discourses on the “problem” of ageing: Consumerized solutions. Discour. Commun. 1, 37–61. 10.1177/1750481307071984

[B14] Craton L. G. Lantos G. P. (2011). Attitude toward the advertising music: An overlooked potential pitfall in commercials. J. Consum. Mark. 28, 396–411. 10.1108/07363761111165912

[B15] Dalmer N. Ellison K. Katz S. Marshall B. (2022). Aging, embodiment and datafication: Dynamics of power in digital health and care technologies. Int. J. Ageing Later Life. 15, 77–101. 10.3384/ijal.1652-8670.3499

[B16] Einsend M. (2022). Older people in advertising. J. Adv. 51, 308–322. 10.1080/00913367.2022.2027300

[B17] Elsaesser T. Hagener M. (2010). Film Theory: An Introduction Through the Senses. New York, NY: Routledge.

[B18] Essence (2020). Essence Care@Home Video [Video]. Available online at: https://www.youtube.com/watch?v=NRQ4f5mT4Pg (accessed July 31, 2022).

[B19] First A. Ramer-Biel S. (2018). “This is not the time and place to grow old”: ageism in advertising for third-age housing. Howard J. Commun. 29, 1–17. 10.1080/10646175.2017.1300963

[B20] Gentry J. W. Mittelstaedt R. A. (2017). The rapidly aging world: Implications for marketing. Global Busin. Rev. 3S, 1S−18S. 10.1177/0972150917693136

[B21] Gilleard C. Higgs P. (2010). Aging without agency: Theorizing the fourth age. Aging Ment Health. 2, 121–128. 10.1080/13607860903228762 20336545

[B22] Gilleard C. J. Higgs P. (2000). Cultures of Ageing: Self, Citizen, and the Body. New York, NY: Prentice Hall.

[B23] Gilleard C. J. Higgs P. (2022). Agents or actants: What technology might make of later life?, in Socio-Gerontechnology: Interdisciplinary critical Studies of Ageing and Technology, eds Peine A. Marshall B.L. Martin W. Neven L. (London: Routledge), 99–111.

[B24] Graakjær N. J. (2015). Analyzing Music in Advertising: Television commercials and Consumer Choice. New York, NY: Routledge.

[B25] Graham M. E. (2016). The voices of Iris: cinematic representations of the aged woman and Alzheimer's disease in Iris (2001). Dementia. 15, 1171–1183. 10.1177/1471301214556133 25370076

[B26] Herget A. Bötzl F. (2021). Sounds like respect. The impact of background music on the acceptance of gay men in audio-visual advertising. Front. Psychol. 12, 1–8. 10.3389/fpsyg.2021.645533 33967905 PMC8100044

[B27] Higgs P. Gilleard C. (2022). Techno-fixes for an ageing society. Aging Mental Health. 26, 1303–1305. 10.1080/13607863.2021.2008308 34823393

[B28] Ivan L. Cutler S. J. (2021). Ageism and technology: The role of internalized stereotypes. Univ. Toronto Quart. 90, 127–139. 10.3138/utq.90.2.05

[B29] Ivan L. Loos E. Tudorie G. (2020). Mitigating visual ageism in digital media: designing for dynamic diversity to enhance communication rights for senior citizens. Societies.10, 1–13. 10.3390/soc,10040076

[B30] JB Hi-Fi Official (2020). Sofihub Home @ JB Hi-Fi [Video]. Available online at: https://www.youtube.com/watch?v=UzZvvJh2a5w (accessed July 31, 2022).

[B31] Katz S. (2015). Ageing, risk and the falling body, in Routledge Handbook of Cultural Gerontology, eds Twigg J. Martin W. (New York, NY: Routledge). 165–172.

[B32] Kenalemang L. M. (2021). Visual ageism and the subtle sexualization of older celebrities in L'Oréal's advert campaigns: a multimodal critical discourse analysis. Ageing Soc. 42, 1–18. 10.1017/S0144686X20002019

[B33] Kress G. Van Leeuwen T. (2020). Reading Images: The Grammar of Visual Design. London: Routledge.

[B34] Lamb E. Gentry J. (2013). The denial of aging in American advertising: Empowering or disempowering. Aging Soc. Int. J. 2, 35–47. 10.18848/2160-1909/CGP/v02i04/35221

[B35] Loos E. Ivan L. (2018). Visual ageism in the media, in Contemporary Perspectives on Ageism, eds Ayalon L. Tesch-Romer C. (Cham: Springer), 163–176.

[B36] Low S. Maguire M. (2019). Spaces of Security: Ethnographies of Securityscapes, Surveillance, and Control. New York, NY: NYU Press.

[B37] Machin D. Van Leeuwen T. (2016). Sound, music and gender in mobile games. Gender Language. 10, 412–432. 10.1558/genl.v10i3.32039

[B38] Marshall B. L. Dalmer N. K. Katz S. Loos E. López Gómez D. (2022). Digitization of aging-in-place: an international comparison of the value-framing of new technologies. Societies. 12, 1–14. 10.3390/soc12020035

[B39] Neven L. Peine A. (2017). From triple win to triple sin: how a problematic future discourse is shaping the way people age with technology. Societies. 7, 1–11. 10.3390/soc7030026

[B40] Peine A. Marshall B. L. Martin W. Neven L. (2021). Socio-gerontechnology: Key themes, future agendas, in Socio-Gerontechnology: Interdisciplinary Critical Studies of Ageing and Technology, eds Peine A. Marshall B.L. Martin W. Neven L. (London: Routledge), 1–23.

[B41] Peine A. Neven L. (2021). The co-constitution of ageing and technology: a model and agenda. Ageing Soc. 41, 2845–2866. 10.1017/S0144686X20000641

[B42] Peine A. Rollwagen I. Neven L. (2014). The rise of the ‘innosumer' – Rethinking older technology users. Technol. Forecast. Soc. Change. 82, 199–214. 10.1016/j.techfore.2013.06.013

[B43] Pirzada P. Wilde A. Doherty G. H. Harris-Birtill D. (2022). Ethics and acceptance of smart homes for older adults. Inform. Health Soc. Care. 47, 10–37. 10.1080/17538157.2021.1923500 34240661

[B44] Rowe J. W. Kahn R. L. (2015). Successful aging 2.0: conceptual expansions for the 21st Century. J. Gerontol. Series B. 70, 593–596. 10.1093/geronb/gbv025 25878054

[B45] Sánchez-Porras M. J. Rodrigo E. M. (2017). Emotional benefits of coca-cola advertising music. Procedia Soc. Behav. Sci. 237, 1444–1448. 10.1016/j.sbspro.2017.02.227

[B46] Tagg P. (2013). Music's Meanings: A Modern Musicology for Non-Musos. New York, NY: The Mass Media Music Scholars' Press.

[B47] Tan S. Spackman M. P. Bezdek M. A. (2007). Viewers' interpretations of film characters' emotions: effects of presenting film music before or after a character is shown. Music Percept. 25, 135–152. 10.1525/mp.2007.25.2.135

[B48] Tural E. Lu D. Cole D. A. (2021). Safely and actively aging in place: Older adults' attitudes and intentions toward smart home technologies. Gerontol. Geriatric Med. 7, 1–15. 10.1177/23337214211017340 34095352 PMC8142240

[B49] Urban M. (2021). Topographies of ageing: A new materialist analysis of ageing-in-place, in Socio-Gerontechnology: Interdisciplinary Critical Studies of Ageing and Technology, eds. Peine A. Marshall B.L. Martin W. Neven L. (London: Routledge), 56–69.

[B50] Van Hees S. Wanka A. Horstman K. (2021). Making and unmaking ageing-in-place: towards a co-constructive understanding of ageing and place, in Socio-Gerontechnology: Interdisciplinary Critical Studies of Ageing and Technology, eds Peine A. Marshall B.L. Martin W. Neven L. (London: Routledge), 133–146.

[B51] Van Hoof J. Kort H. S. Rutten P. G. Duijnstee M. S. (2011). Ageing-in-place with the use of ambient intelligence technology: perspectives of older users. Int J Med Inform. 80:5, 310–331. 10.1016/j.ijmedinf.2011.02.010 21439898

[B52] Van Leeuwen T. (1998). Music and ideology: Notes toward a sociosemiotics of mass media music. Popular Music Soc. 22, 25–54. 10.1080/03007769808591717

[B53] Van Leeuwen T. (1999). Speech, Music, Sound. London: Macmillan Press.

[B54] Van Leeuwen T. (2012). The critical analysis of musical discourse. Crit. Disc. Studies. 9, 319–328. 10.1080/17405904.2012.713204

[B55] Vermeer Y. Higgs P. Charlesworth G. (2019). Marketing of surveillance technology in three ageing countries. Quality Ageing Older Adults. 28, 20–33. 10.1108/QAOA-03-2018-0010

[B56] Vermeer Y. Higgs P. Charlesworth G. (2020). Selling surveillance technology: semiotic themes in advertisements for ageing-in-place with dementia. Soc. Semiotics. 10.1080/10350330.2020.1767399. [Epub ahead of print].

[B57] WHO (2022a). Ageing. Available online at: https://www.who.int/health-topics/ageing#tab=tab_1 (accessed June 2, 2022)

[B58] WHO (2022b). UN Decade of Healthy Ageing: 2021-2030. Available online at: https://www.who.int/initiatives/decade-of-healthy-ageing (accessed June 4, 2022).

[B59] Ylänne V. (2015). Representations of ageing in the media, in Routledge Handbook of Cultural Gerontology, eds Twigg J. Martin E. (New York, NY: Routledge), 369–375.

